# Smoking influence in Takotsubo syndrome: insights from an international cohort

**DOI:** 10.3389/fcvm.2023.1282018

**Published:** 2023-11-20

**Authors:** Iván J. Núñez-Gil, Francesco Santoro, Ravi Vazirani, Giuseppina Novo, Emilia Blanco-Ponce, Luca Arcari, Aitor Uribarri, Luca Cacciotti, Federico Guerra, Jorge Salamanca, Beatrice Musumeci, Oscar Vedia, Enrica Mariano, Clara Fernández-Cordón, Pasquale Caldarola, Roberta Montisci, Natale Daniele Brunetti, Ibrahim El-Battrawy, Mohammad Abumayyaleh, Ibrahim Akin, Ingo Eitel, Thomas Stiermaier

**Affiliations:** ^1^ Cardiology Department, Hospital Clínico San Carlos, Madrid, Spain; ^2^ Faculty of Biomedical and Health Sciences, Universidad Europea de Madrid, Madrid, Spain; ^3^ Department of Medical and Surgical Sciences, University of Foggia, Foggia, Italy; ^4^ Cardiology Unit, Department of Health Promotion, Mother and Child Care, Internal Medicine and Medical Specialties, University of Palermo, Palermo, Italy; ^5^ Cardiology Department, Hospital Universitario Arnau de Vilanova, Lérida, Spain; ^6^ Institute of Cardiology, Madre Giuseppina Vannini Hospital, Rome, Italy; ^7^ Servicio de Cardiología, Hospital Universitari Vall d´Hebron, Barcelona, Spain; ^8^ Department of Cardiology, Hospital de Vall d'Hebron, Barcelona, Spain; ^9^ CIBER-CV, Barcelona, Spain; ^10^ Cardiology and Arrhythmology Clinic, University Hospital “Umberto I - Lancisi - Salesi”, Marche Polytechnic University, Ancona, Italy; ^11^ Department of Cardiology, Instituto de Investigación Sanitaria Princesa (IIS-IP), Hospital Universitario de La Princesa, Madrid, Spain; ^12^ Cardiology, Department of Clinical and Molecular Medicine, Sant'Andrea Hospital, Sapienza University, Rome, Italy; ^13^ Cardiology Department, Tor Vergata University, Rome, Italy; ^14^ Department of Cardiology, Hospital Universitario Gregorio Marañón, Madrid, Spain; ^15^ Department of Cardiology, San Paolo Hospital, Asl Bari, Italy; ^16^ Clinical Cardiology, AOU Cagliari, Department of Clinical Sciences and Public Health, University of Cagliari, Cagliari, Italy; ^17^ Department of Cardiology and Angiology, Bergmannsheil University Hospitals, Ruhr University of Bochum, Bochum, Germany; ^18^ Department of Cellular and Translational Physiology and Institut für Forschung und Lehre (IFL), Molecular and Experimental Cardiology, Institute of Physiology, Ruhr-University Bochum, Bochum, Germany; ^19^ First Department of Medicine, University Medical Center Mannheim, Mannheim, Germany; ^20^ Medical Clinic II, University Heart Center Lübeck, Lübeck, Germany; ^21^ German Center for Cardiovascular Research (DZHK), Partner site Hamburg - Kiel - Lübeck, Lübeck, Germany

**Keywords:** Takotsubo syndrome, prognosis, smoking habit, mortality, registry, apical ballooning

## Abstract

**Aims:**

To assess the influence of tobacco on acute and long-term outcomes in Takotsubo syndrome (TTS).

**Methods:**

Patients with TTS from the international multicenter German Italian Spanish Takotsubo registry (GEIST) were analyzed. Comparisons between groups were performed within the overall cohort, and an adjusted analysis with 1:1 propensity score matching was conducted.

**Results:**

Out of 3,152 patients with TTS, 534 (17%) were current smokers. Smoker TTS patients were younger (63 ± 11 vs. 72 ± 11 years, *p* < 0.001), less frequently women (78% vs. 90%, *p* < 0.001), and had a lower prevalence of hypertension (59% vs. 69%, *p* < 0.01) and diabetes mellitus (16% vs. 20%, *p* = 0.04), but had a higher prevalence of pulmonary (21% vs. 15%, *p* < 0.01) and/or psychiatric diseases (17% vs. 12%, *p* < 0.01). On multivariable analysis, age less than 65 years [OR 3.85, 95% CI (2.86–5)], male gender [OR 2.52, 95% CI (1.75–3.64)], history of pulmonary disease [OR 2.56, 95% CI (1.81–3.61)], coronary artery disease [OR 2.35, 95% CI (1.60–3.46)], and non-apical ballooning form [OR 1.47, 95% CI (1.02–2.13)] were associated with smoking status. Propensity score matching (PSM) 1:1 yielded 329 patients from each group. Smokers had a similar rate of in-hospital complications but longer in-hospital stays (10 vs. 9 days, *p* = 0.01). During long-term follow-up, there were no differences in mortality rates between smokers and non-smokers (5.6% vs. 6.9% yearly in the overall, *p* = 0.02, and 6.6%, vs. 7.2% yearly in the matched cohort, *p* = 0.97).

**Conclusions:**

Our findings suggest that smoking may influence the clinical presentation and course of TTS with longer in-hospital stays, but does not independently impact mortality.

## Introduction

Takotsubo syndrome (TTS), also known as stress-induced cardiomyopathy or broken heart syndrome, is a rare but potentially life-threatening condition characterized by acute and reversible ventricular dysfunction (1–4). While it clinically can mimic an acute coronary syndrome (ACS), the etiology of TTS remains unknown and is frequently linked with stressful situations (mainly negative but also positive).

Similar to ACS, recent research has identified several differential potential risk factors including different metabolomics (5).

On the other hand, tobacco smoking habit has been associated with the development of several conditions and increased mortality, contributing to more than 7 million deaths every year and being a main cause of preventable diseases worldwide (6). Global statistics indicate that there are approximately 1.1 billion active smokers and that 80% of them live in low- and middle-income countries (6). However, the potential influence of smoking on TTS is not fully understood due to limited available data (7). Given the potential risks associated with smoking and cardiovascular diseases, and the fact that women with TTS have been deemed to report a higher prevalence of lifetime smoking than healthy women volunteers (8), the present study aimed to undertake an analysis of the impact of smoking on patients with TTS included in the large, multicentric and international German Italian Spanish Takotsubo (GEIST) registry, to provide a more comprehensive picture of disease characteristics and outcomes in this subset of patients.

## Methods

This study included data from 3,152 consecutive patients enrolled in the GEIST registry [NCT04361994] as of November 2022. As previously reported, the inclusion of patients in the registry was performed by fulfilling the following TTS diagnostic criteria: (1) transient segmental wall motion abnormalities of the left or right ventricle, extending beyond a single epicardial vascular distribution; (2) absence of any potential culprit coronary artery disease; (3) new and reversible electrocardiography abnormalities; (4) elevated cardiac troponin levels; and (5) recovery of ventricular systolic function at follow-up unless previously deceased (9).

**Figure 1:**
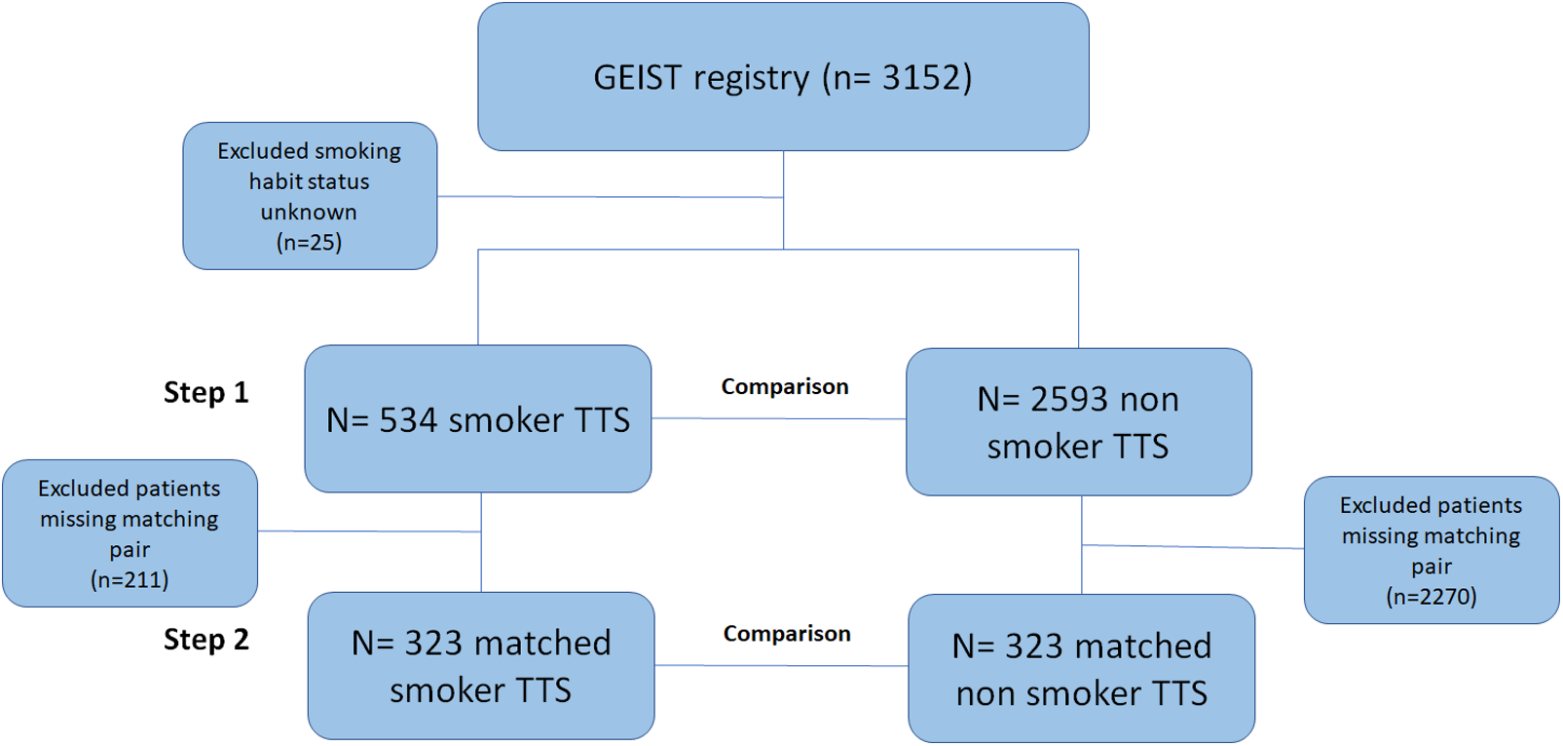
The study consisted of an analysis of the international multicenter GEIST registry. The diagram depicts the study design, for which comparisons were performed between smoker and non-smoker TTS patients both in the overall (step 1) and matched (step 2) cohorts. GEIST, German Italian Spanish Takotsubo; TTS, Takotsubo syndrome.

Coronary artery disease was defined as the presence of coronary calcifications, atherosclerosis, plaques, and/or stenosis, with stenosis >50% of the lumen diameter in arteries with a diameter amenable to percutaneous intervention in previous admissions (9). TTS secondary forms, as previously defined (10), were considered when a physical trigger or emotional plus physical triggers were present. Primary forms were those without triggers or with an isolated identifiable emotional trigger (10, 11). Right ventricular involvement was considered as such when any abnormality was demonstrated in the echocardiogram at that level and included in the clinical report. Left ventricular tract (LVOT) obstruction was considered when a ≥30 mmHg gradient was demonstrated in the echocardiogram or after an invasive direct measurement. Left ventricular thrombus was considered when a compatible image was discovered in any imaging technique. For the purposes of the present study, we provided a stepwise comparison between current smoker TTS patients (at the admission time) and non-smoker TTS patients within the study sample (Figure 1).

Initially (Step 1), the comparison was made for smoker and non-smoker patients within the overall population, after excluding those patients with current smoking habit status not disclosed or unknown (*n* = 25).

Afterward (Step 2), a propensity score matching (PSM) analysis was used to identify subgroups of smokers and non-smoker TTS patients with a 1:1 nearest neighbor matching. After univariate assessment, PSM variables included age, sex, hypertension, diabetes, malignancies, coronary artery disease, atrial fibrillation, and any pulmonary or psychiatric disease. Match tolerance was set at 0.02, without replacement. The matched cohort consisted of 329 pairs of TTS patients for which additional comparison was carried out.

Demographic and clinical characteristics as well as the presence and type of the preceding stressful trigger, ballooning patterns, in-hospital complications, and outcome were recorded at admission, as previously described (2, 9, 12). Recovery of left ventricular systolic function was documented 1–6 months after the acute event in all surviving patients. Long-term outcome was tested by outpatient visits, medical records, or phone interviews. All patients were managed in accordance with the Declaration of Helsinki and signed an informed consent for the processing of personal data for scientific research purposes. The primary outcome was all-cause fatality.

### Statistical analyses

Categorical variables were compared using a Chi-square analysis or Fisher's exact test as appropriate. Continuous variables were presented as mean ± standard deviation. The Student's *T*-test for independent samples was used to compare continuous variables. Univariable and multivariable logistic regression analyses were used to calculate estimated ORs and 95% CIs for factors associated with smoking habits in patients with TTS. Collinearity was tested and dismissed considering variance inflation factors (VIF) as <5. Cox regression analysis models were carried out to assess factors independently associated with long-term mortality in the overall cohort. Standardized mean differences (SMD) were provided to depict effect size in the overall and the matched cohort after PSM. Kaplan–Meier curves and log-rank test were used to assess survival function at follow-up in both the overall and matched cohort. All data were analyzed with SPSS software version 24.0 (SPSS Inc., Chicago, Illinois, USA). A two-tailed *p*-value <0.05 was considered significant.

## Results

### Clinical characteristics

**Figure 2:**
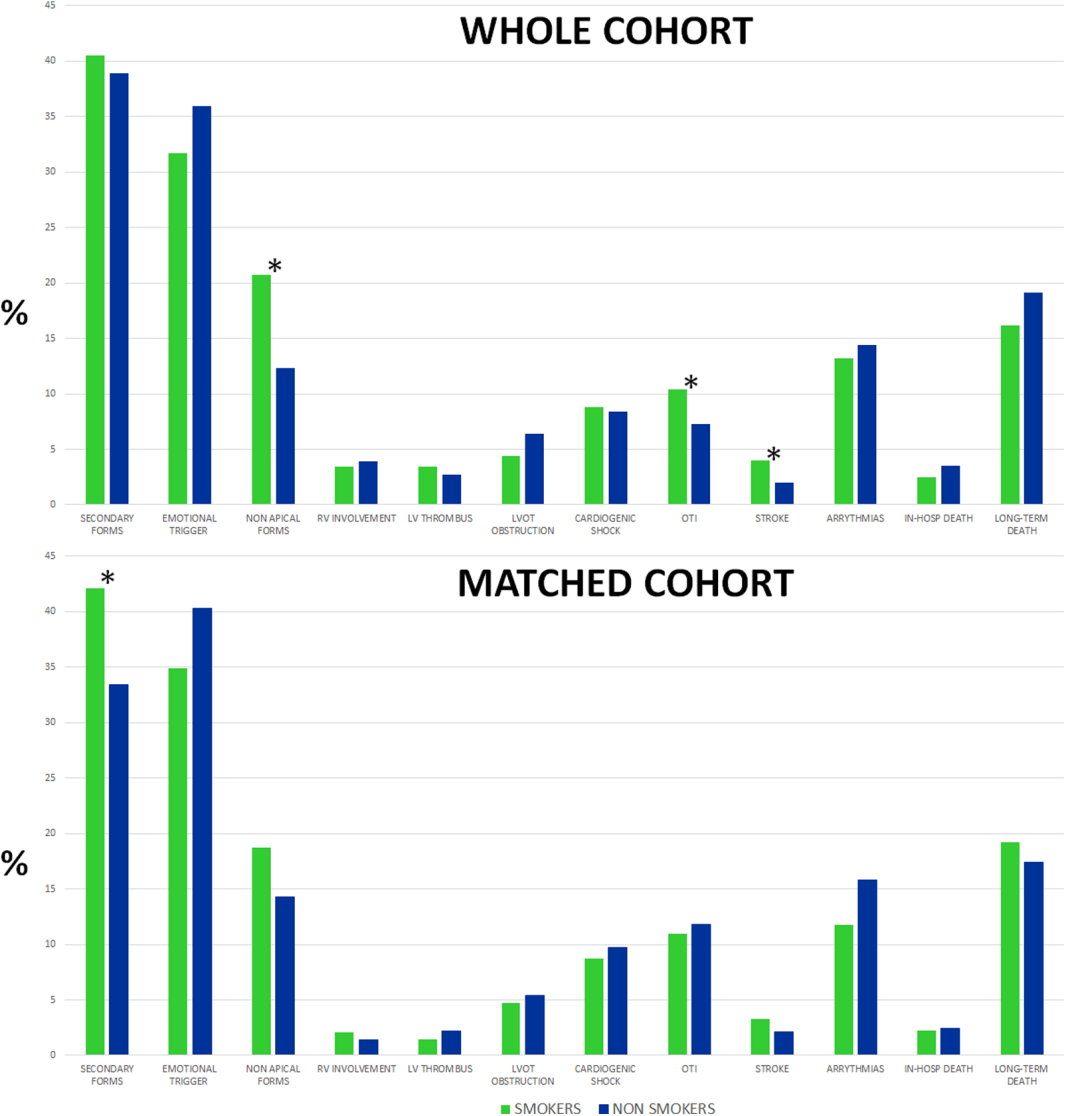
TTS presentation profile, in-hospital complications, and mortality were dichotomized by smoking habit in the overall population (top) and matched cohort (bottom). *Statistical differences of clinical features (*p* < 0.05). TTS, Takotsubo syndrome.

Baseline demographic, clinical characteristics, and outcomes within the overall and matched cohorts stratified by smoking habit are provided in Table 1 with events summarized in Figure 2. Of the 3,127 patients included in the present analysis from the GEIST registry, 534 (17%) were smokers.

**Table 1:** Baseline characteristics of patients within the overall and matched cohorts.

Variable	Overall cohort		*p*-values	SMD	Matched cohort		*p*-values	SMD
	Smoker TTS (*n* = 534)	Non-smoker TTS (*n* = 2,593)			Smoker TTS (*n* = 329)	Non-smoker TTS (*n* = 329)		
Age (years)^a^	63 ± 11	72 ± 11	**<0** **.** **001**	0.723	64 ± 11	64 ± 13	0.464	0.058
Comorbidities								
Hypertension (%)^a^	317/533 (59)	1,800/2,590 (69)	**<0** **.** **001**	0.214	191/323 (51)	184/323 (50)	0.577	−0.043
Diabetes mellitus (%)^a^	88/533 (16)	523/2,586 (20)	**0** **.** **049**	0.093	60/323 (52)	55/323 (48)	0.607	−0.041
Dyslipidemia (%)	219/481 (45)	1,038/2,464 (42)	0.167	−0.068	132/276 (48)	86/242 (35)	0.003	−0.257
Sex female (%)^a^	418/534 (78)	2,332/2,591 (90)	**<0** **.** **001**	−0.361	259/323 (80)	269/323 (83)	0.309	−0.077
Pulmonary disease (%)^a^	122/479 (25)	363/2,350 (15)	**<0** **.** **001**	−0.266	80/323 (25)	76/323 (23)	0.713	−0.028
Malignancies (%)^a^	58/492 (12)	325/2,285 (14)	0.155	0.071	33/323 (10)	25/323 (8)	0.271	−0.081
Psychiatric disease (%)^a^	74/434 (17)	258/2,146 (12)	**0** **.** **004**	−0.150	50/323 (8)	50/323 (8)	1.000	<0.001
Coronary artery disease (%)^a^	71/481 (15)	200/2,309 (9)	**<0** **.** **001**	−0.206	50/323 (15)	62/323 (19)	0.212	0.099
Clinical presentation								
Chest pain (%)	287/495 (58)	1,468/2,488 (59)	0.673	0.021	194/322 (60)	188/319 (59)	0.735	−0.026
Dyspnea (%)	180/495 (36)	827/2,498 (33)	0.161	−0.068	117/321 (36)	102/321 (32)	0.212	−0.098
Stressful trigger (%)	370/531 (70)	1,853/2,587 (72)	0.366	0.043	242/322 (75)	230/323 (71)	0.258	−0.086
Emotional (%)	178/531 (33)	958/2,587 (37)	0.126	0.072	115/322 (41)	132/323 (21)	0.178	0.113
Physical (%)	198/531 (37)	925/2,587 (36)	0.503	−0.031	131/322 (40)	191/323 (31)	0.013	−0.197
Admission electrocardiographic findings								
Atrial fibrillation (%)^a^	47/456 (10)	357/2,251 (16)	**0** **.** **002**	0.155	33/323 (10)	42/323 (56)	0.269	0.087
ST-changes (%)	343/438 (78)	1,705/2,303 (74)	0.059	−0.098	223/275 (81)	193/236 (81)	0.842	0.017
Admission echocardiographic findings								
Apical ballooning (%)	383/483 (79)	2,005/2,287 (88)	**<0** **.** **001**	0.242	226/278 (81)	269/314 (86)	0.151	0.113
Mid-ventricular ballooning (%)	83/482 (17)	245/2,279 (11)	**<0** **.** **001**	−0.199	46/277 (17)	43/306 (14)	0.392	−0.069
Basal ballooning (%)	17/478 (4)	36/2,227 (2)	**0** **.** **004**	−0.144	6/305 (2)	7/274 (2)	0.932	0.006
Focal (%)	3/440 (0.7)	5/2,039 (0.2)	0.143	−0.077	2/267 (1)	0/298 (1)	0.134	−0.117
LVEF (%)	41 ± 10	40 ± 10	0.112	−0.077	41 ± 10	41 ± 10	0.437	0.061
In-hospital course and treatment								
In-hospital complications	118/534 (22)	530/2,593 (20)	0.389	−0.014	72/323 (22)	65/323 (20)	0.500	−0.052
Pulmonary edema (%)	43/520 (8)	220/2,560 (9)	0.809	0.011	28/322 (9)	23/319 (7)	0.487	−0.055
Cardiogenic shock (%)	47/534 (9)	217/2,586 (8)	0.757	−0.014	28/323 (9)	31/320 (10)	0.655	0.035
Arrhythmias (%)	39/296 (13)	226/1,571 (14)	0.584	0.034	23/195 (12)	28/177 (16)	0.260	0.115
Stroke	18/450 (4)	54/2,299 (2.3)	**0** **.** **045**	−0.103	9/273 (3)	6/283 (2)	0.392	−0.072
Death (%)	12/478 (2)	80/2,271 (3)	0.263	0.055	6/276 (2)	7/288 (2)	0.829	0.016
Catecholamine (%)	52/477 (11)	233/2,374 (10)	0.470	−0.036	34/293 (12)	33/274 (12)	0.871	0.013
OTI (%)	50/480 (10)	177/2,439 (7)	**0** **.** **018**	−0.118	31/283 (11)	30/255 (12)	0.767	0.026
In-hospital stay (days)	10 ± 11	9 ± 9	**0** **.** **017**	−0.123	10 ± 11	8 ± 6	**0** **.** **015**	0.204
Discharge therapy and vital status after follow up								
Aspirin (%)	305/474 (64)	1,376/2,320 (59)	**0** **.** **041**	−0.102	194/296 (65)	164/295 (56)	**0** **.** **013**	−0.203
DAPT (%)	75/339 (22)	280/1,702 (16)	**0** **.** **012**	−0.149	51/205 (25)	34/178 (19)	0.175	−0.141
Oral anticoagulant (%)	78/415 (19)	513/2,148 (24)	**0** **.** **024**	0.120	50/254 (20)	43/268 (16)	0.277	−0.095
Beta-blockers (%)	314/454 (69)	1,594/2,229 (71)	0.314	0.314	225/296 (71)	211/296 (76)	0.192	0.105
Ace-inhibitor/ARB (%)	315/476 (66)	1,613/2,338 (69)	0.228	0.228	201/294 (68)	209/300 (70)	0.732	0.027
Statin (%)	257/474 (54)	1,281/2,308 (55)	0.609	0.609	163/298 (55)	135/290 (47)	**0** **.** **048**	−0.163
Death (%)	79/488 (16)	459/2,407 (19)	0.136	0.074	57/297 (19)	38/218 (17)	0.611	−0.047
Total follow-up (days)	965 ± 3,213	1,026 ± 2,210	0.686	−0.019	1,001 ± 1,268	623 ± 909	**<0** **.** **001**	−0.319

Values are mean ± SD, or *n*/*N* (%). ACE, angiotensin-converting enzyme; ARB, angiotensin II receptor blocker; DAPT, dual antiplatelet therapy; LVEF, left ventricular ejection fraction; OTI, oro-tracheal intubation; SMD, standardized mean difference; TTS, Takotsubo syndrome.

^a^ Variables that were used to calculate the propensity score for matching.

Smoker TTS patients were significantly younger (63 ± 11 vs. 72 ± 11 years, *p* < 0.001), less frequently women (78% vs. 90%, *p* < 0.001), and presented with significantly lower prevalence of some comorbid conditions (hypertension 59% vs. 69%, *p* < 0.001; diabetes mellitus 16% vs. 20%, *p* = 0.049 or admission atrial fibrillation 10% vs. 16%, *p* = 0.002) but had a higher prevalence of pulmonary diseases (21% vs. 15%, *p* < 0.001) or psychiatric diseases (17% vs. 12%, *p* = 0.004), as compared to non-smokers.

On clinical presentation, smoker TTS patients reported a similar dyspnea rate (36% vs. 33%, *p* = 0.16) and similar chest pain percentage compared to non-smokers (58% vs. 59%, *p* = 0.67), which was the main onset complaint. If any stressful trigger was frequent, it was present in a similar percentage between smokers and non-smokers (70% vs. 72%, *p* = 0.366) with a similar distribution of emotional and physical causes.

No significant differences were detected regarding ECG parameters but the ballooning pattern was dissimilar. Smoker TTS patients presented with a lower rate of apical ballooning (79% vs. 88%, *p* < 0.001) and a higher percentage of atypical forms, but overall onset left ventricular ejection fraction was similar (41 ± 10% vs. 40 ± 10%, *p* = 0.11).

On multivariable analysis (Table 2), older age [odds ratio (OR for ≥65 years) 0.26, 95% CI (0.20–0.35)], male sex [OR 2.52, 95% CI (1.75–3.64)], presence of pulmonary disease [OR 2.56, 95% CI (1.81–3.61)], coronary artery disease [OR 2.35, 95% CI (1.60–3.46)], and non-apical ballooning form [OR 1.47, 95% CI (1.02–2.13)] remained independently associated with the smoking status.

**Table 2:** Univariable and multivariable analysis for factors associated with smoking habit in the overall TTS cohort.

Factors associated with smoking habit					
Variable	Univariable		Multivariable		
	OR (95% CI)	*p*-values	OR (95% CI)	*p*-values	VIF
Age (≥65 years)	0.25 (0.20–0.30)	**<0** **.** **001**	0.26 (0.20–0.35)	**<0.001**	1.121
Sex male	2.34 (1.68–3.18)	**<0** **.** **001**	2.52 (1.75–3.64)	**<0.001**	1.014
Hypertension	0.64 (0.53–0.78)	**<0** **.** **001**	NS	NS	1.084
Diabetes	0.78 (0.61–1.00)	**0** **.** **049**	NS	NS	1.060
Dyslipidemia	1.15 (0.94–1.40)	0.167	–	–	–
Pulmonary disease	1.87 (1.48–2.36)	**<0** **.** **001**	2.56 (1.81–3.61)	**<0.001**	1.024
Malignancies	0.81 (0.60–1.09)	0.155	–	–	–
Coronary artery disease	1.83 (1.36–2.44)	**<0** **.** **001**	2.35 (1.60–3.46)	**<0.001**	1.029
Psychiatric disease	1.50 (1.13–1.99)	**0** **.** **004**	NS	NS	1.010
Atrial fibrillation	0.61 (0.44–0.84)	**0** **.** **002**	NS	NS	1.040
Stressful trigger	0.91 (0.74–1.12)	0.366	–	–	–
Onset LVEF (≥55%)	0.94 (0.70–1.25)	0.664	–	–	–
Non-apical ballooning	0.54 (0.42–0.69)	**<0** **.** **001**	1.47 (1.02–2.13)	**0.039**	1.057

LVEF, left ventricular ejection fraction; NS, non-significant; VIF, variance inflation factor.

### Outcome

During the hospital course, in the overall cohort, smokers presented a comparable percentage of complications, except for a higher need for orotracheal intubation and stroke rate (Figure 2). Moreover, smoker TTS patients had longer lengths of hospital stays (10 vs. 9 days, *p* = 0.017) with similar in-hospital mortality (2% vs. 3%, *p* = 0.26). At discharge, smoker TTS patients received a more intense antiplatelet treatment.

After propensity score matching, smoker and non-smoker TTS patients presented no significant differences regarding in-hospital complications, as shown in Table 1 and Figure 2.

During long-term follow-up, mortality rates were 6.7% per patient-year (smokers 5.6% and non-smokers 6.9%, *p* = 0.02) in the overall cohort and 6.8% per patient-year (smokers 6.6%, non-smokers 7.2%, *p* = 0.97) in the matched cohort respectively.

**Figure 3:**
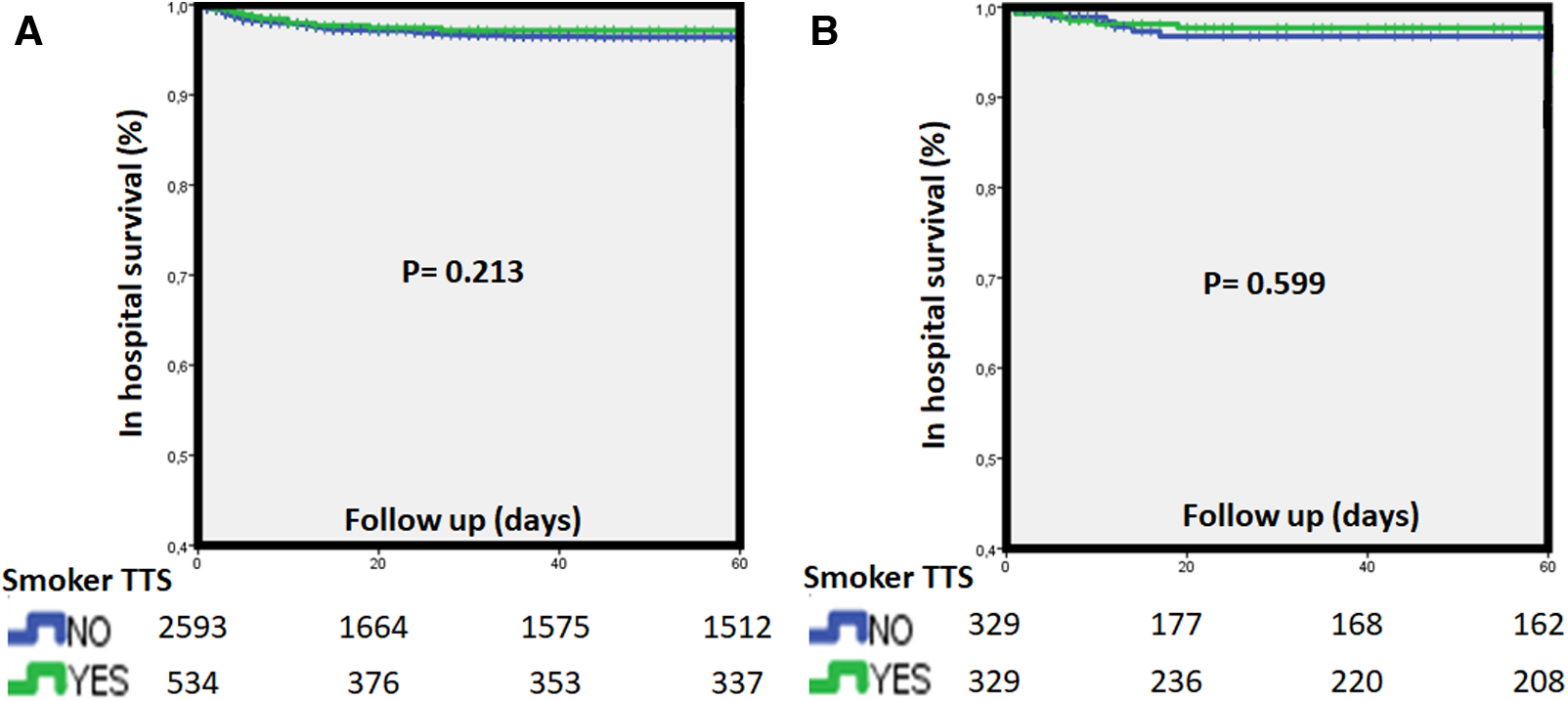
Kaplan-Meier survival curves for in-hospital death (**A**) dichotomized by smoking habit in the overall population (top) and matched cohort (**B**) TTS, Takotsubo syndrome.

**Figure 4:**
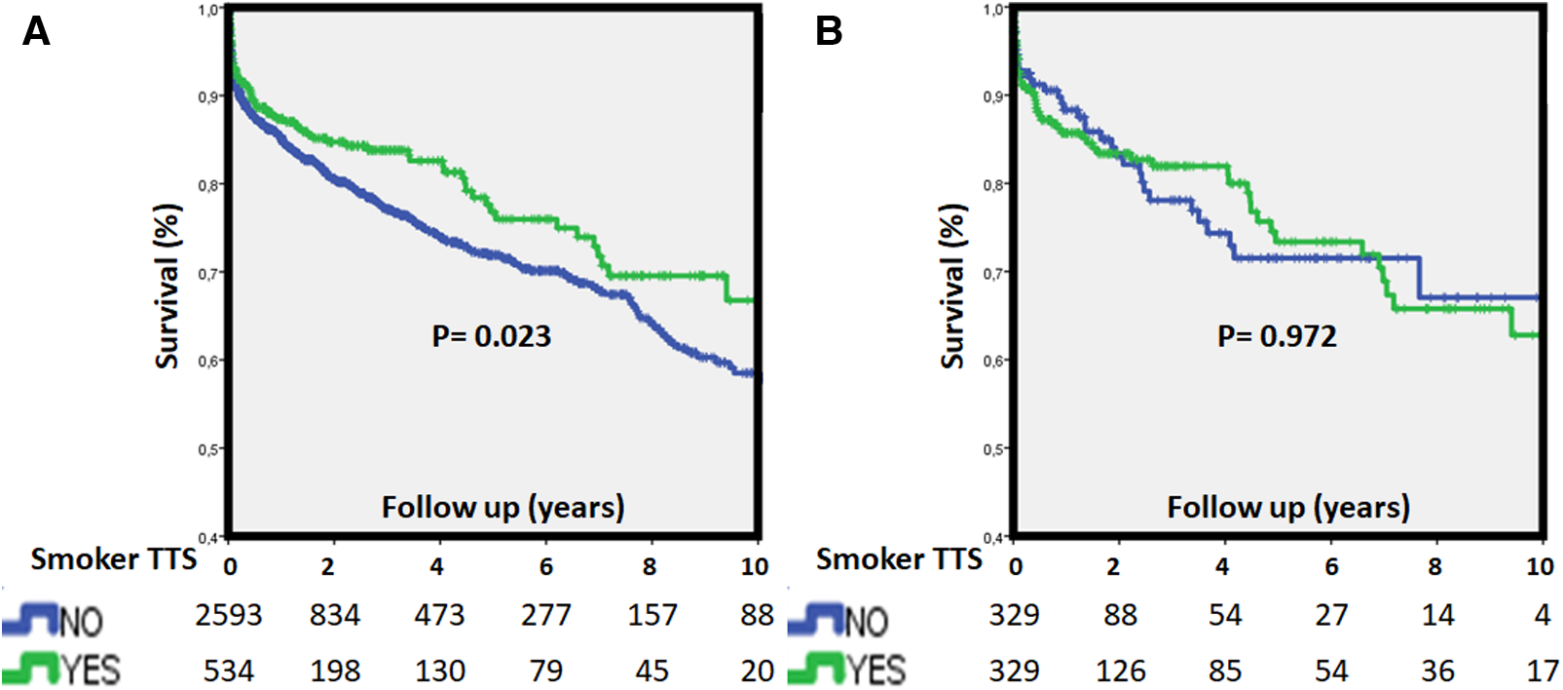
Kaplan-Meier survival curves for long-term (**A**) mortality stratified by smoking habit in the overall population (top) and matched cohort (**B**) long-term worse outcome in non-smokers was detected, albeit there was similar long-term mortality after adjustment. TTS, Takotsubo syndrome.

Smoker TTS patients experienced similar in-hospital mortality rates both in the overall cohort and in the matched cohort (2% vs. 3% and 2% vs. 2%; *p* = 0.263 and *p* = 0.829, respectively). Assessing long-term mortality rate after the complete follow-up, smoker TTS presented numerically less mortality in the overall cohort but not in the matched cohort (16% vs. 19%, *p* = 0.14 and 19% vs. 17% *p* = 0.61, respectively). Kaplan-Meier actuarial survival curves with in-hospital and long-term mortality in the study cohorts are displayed in Figures 3, 4.

In the multivariable analysis (Table 3), smoker habit was not shown to be an independent predictor of in-hospital mortality or long-term mortality, in line with PSM analysis. In the present study, independent predictors of long-term mortality were age [hazard ratio (HR) 1.17 per 5 years increase; 95% CI (1.10–1.25); *p* < 0.001], male sex [HR 1.63; 95% CI (1.18–2.24); *p* = 0.003], diabetes [HR 1.60; 95% CI (1.24–2.05); *p* < 0.001], pulmonary disease [HR 1.36; 95% CI (1.02–1.82); *p* = 0.039], malignancies [HR 1.38; 95% CI (1.03–1.85); *p* = 0.029], absence of onset ST changes [HR 2.32; 95% CI (1.85–2.50); *p* < 0.001], secondary TTS forms [HR 2.40; 95% CI (1.89–3.05); *p* < 0.001], and LVEF at admission [HR 1.15 per 5 points decrease; 95% CI (1.09–1.22); *p* < 0.001].

**Table 3:** Univariable and multivariable Cox regression analysis for factors associated with in-hospital mortality (top).

Variable	Univariable		Multivariable		
	HR (95% CI)	*p*-values	HR (95% CI)	*p*-values	VIF
In-hospital mortality					
Age (per 5-year increase)	1.25 (1.12–1.38)	<0.001	1.25 (1.08–1.45)	**0.002**	1.163
Male sex	2.66 (1.66–4.27)	<0.001	1.83 (0.95–3.52)	0.069	1.043
Hypertension	1.21 (0.77–1.92)	0.404	–	–	
Dyslipidemia	0.86 (0.55–1.36)	0.528	–	–	
Diabetes	2.06 (1.33–3.18)	**0** **.** **002**	NS	NS	1.047
Current smoking	0.68 (0.37–1.25)	0.216	–	–	
Pulmonary disease	2.07 (1.28–3.35)	**0** **.** **005**	NS	NS	1.118
Malignancies	2.11 (1.30–3.43)	**0** **.** **003**	NS	NS	1.038
Coronary artery disease	1.09 (0.52–2.27)	0.819	–	–	
Atrial fibrillation	2.70 (1.68–4.33)	**<0** **.** **001**	NS	NS	1.038
Psychiatric disease	1.04 (0.53–2.04)	0.899	–	–	
Chest pain	0.39 (0.25–0.60)	**<0** **.** **001**	NS	NS	1.196
Dyspnea	2.50 (1.60–3.80)	**<0** **.** **001**	1.80 (1.03–3.14)	**0.038**	1.139
ST changes	1.31 (0.70–2.43)	0.397	–	–	
Secondary TTS form	2.74 (1.80–4.16)	**<0** **.** **001**	NS	NS	1.163
Apical ballooning	2.71 (1.10–6.70)	**0** **.** **030**	NS	NS	1.084
LVEF (per 5-point increase)	0.68 (0.61–0.76)	**<0.001**	0.66 (0.57–0.77)	**<0.001**	1.110
LVOT obstruction	0.91 (0.22–3.76)	0.893	–	–	
RV involvement	0.49 (0.07–3.57)	0.485	–	–	
Long-term mortality					
Age (per 5-year increase)	1.24 (1.19–1.30)	**<0** **.** **001**	1.17 (1.10–1.25)	**<0.001**	1.299
Male sex	1.87 (1.50–2.35)	**<0** **.** **001**	1.63 (1.18–2.24)	**0.003**	1.081
Hypertension	1.36 (1.12–1.65)	**0** **.** **002**	NS	NS	1.181
Dyslipidemia	0.94 (0.84–1.20)	0.936	–	–	
Diabetes	1.72 (1.43–2.08)	**<0** **.** **001**	1.60 (1.24–2.05)	**<0.001**	1.105
Current smoking	0.76 (0.60–0.96)	**0** **.** **023**	0.75 (0.53–1.07)	0.109	1.137
Pulmonary disease	1.80 (1.47–2.22)	**<0** **.** **001**	1.36 (1.02–1.82)	**0.039**	1.180
Malignancies	2.04 (1.65–2.52)	**<0** **.** **001**	1.38 (1.03–1.85)	**0.029**	1.071
Coronary artery disease	1.41 (1.07–1.87)	**0** **.** **015**	NS	NS	1.075
Atrial fibrillation	1.89 (1.53–2.33)	**<0** **.** **001**	NS	NS	1.098
Psychiatric disease	1.08 (0.82–1.41)	0.584	–	–	
Chest pain	0.57 (0.48–0.68)	**<0** **.** **001**	NS	NS	1.290
Dyspnea	1.51 (1.26–1.80)	**<0** **.** **001**	NS	NS	1.219
ST changes	0.67 (0.55–0.82)	**<0** **.** **001**	0.43 (0.40–0.54)	**<0.001**	1.109
Secondary TTS form	2.61 (2.20–3.10)	**<0** **.** **001**	2.40 (1.89–3.05)	**<0.001**	1.230
Apical ballooning	1.90 (1.40–2.56)	**<0** **.** **001**	1.40 (0.95–2.07)	0.086	1.128
LVEF (per 5-point increase)	0.83 (0.79–0.86)	**<0** **.** **001**	0.87 (0.82–0.92)	**<0.001**	1.112
LVOT obstruction	1.27 (0.80–2.03)	0.310	–	–	
RV involvement	1.42 (0.89–2.28)	0.145	–	–	

Univariable and multivariable Cox regression analysis for factors associated with long-term mortality (bottom). LVEF, Left ventricular ejection fraction; LVOT, left ventricular outflow tract; RV, right ventricle; NS, non-significant (*p*-value >0.10); VIF, variance inflation factor. All conditions and ECG/echocardiographic findings are referred to the acute phase. Smoking habit is displayed for reference purposes and was not statistically significant.

## Discussion

To the best of our knowledge, this research provides the largest study addressing the impact of tobacco on patients with TTS. The main findings from this international TTS registry are as follows: (1) Smoking habit has a prevalence of approximately 17% within the overall GEIST population and is independently associated with younger age, male sex, pulmonary and coronary disease, and a higher percentage of atypical TTS forms (non-apical); (2) In-hospital complications are pretty similar between smokers and non-smokers, although the length of hospital stay is higher in the smoking group in both the overall and matched cohorts; and (3) Long-term mortality after the acute phase is not significantly different between smokers and non-smokers in the matched cohort.

Tobacco use is a well-documented major risk factor for cardiovascular diseases (6) such as coronary artery disease, myocardial infarction, peripheral artery disease, and hypertension. It also increases the risk of other cardiovascular complications such as deep vein thrombosis, pulmonary embolism, and arrhythmias, including atrial fibrillation. Additionally, tobacco use significantly enhances the likelihood of ischemic stroke and malignancies among other diseases. Quitting smoking is crucial in reducing the risk of developing cardiovascular complications.

The pathophysiology of TTS is still unclear and likely depends on various factors, including different diseases with different causes and outcomes. It has been associated with several stressful triggers, interestingly even in positive situations (“happy heart syndrome”) (13). Recently, Li et al. identified four TTS patient clusters (14), with prognostic relevance, using latent class analysis in an administrative database. One of these clusters, where all patients had chronic obstructive pulmonary disease (COPD) and many were smokers (45.8%), had the highest in-hospital mortality (3.4%). In addition to the fact that COPD and smoking share pathophysiological mechanisms, tobacco use can also disrupt the balance of the autonomic nervous system and increase plasma catecholamine levels (15, 16). Some studies have suggested that sympathetic nervous system activation and higher plasma catecholamine levels are associated with poor prognosis in patients with TTS (17). In fact, TTS has been described to happen during severe dyspnea in COPD, a specific phenotype called “bronchogenic” TTS, with frequent atypical presentation (18). Moreover, patients with pulmonary disease are more prone to develop acute respiratory failure, which, together with TTS could worsen the prognosis (14). In our smoking cohort, despite being younger, we found a higher raw incidence of orotracheal intubation (OTI) and atypical forms reflecting this point.

Regarding the clinical presentation, Zaghol et al. described a single-center series of 264 patients with TTS showing that smokers had a different clinical profile, consistent with our study. Indeed, patients were younger (65.6 vs. 68.5 years), there were more men (16.2% vs. 6.8%), and they had a higher prevalence of pulmonary diseases-COPD (30.8% vs. 6.7%), as well as presented with dyspnea (50.0% vs. 38.1%), requiring more frequently mechanical ventilation, with more atypical forms, despite similar ejection fraction and wall motion abnormality distribution (7). Length of intensive unit and hospitalization stay were also higher in smokers (7), as in the present study.

Yet, in our series, once PSM was adjusted by comorbidities, including age, sex, or pulmonary disease, the differences in mechanical ventilation disappeared although a numerical trend of non-apical forms was maintained.

Redfors et al. reported a Swedish nationwide comparison among TTS, STEMI, and NSTEMI with SCAAR data. This study found smoking habit as an independent predictor of adverse clinical outcomes regarding acute heart failure (HR 2.11 95% CI 1.44–3.08), 30-day mortality (HR 2.29 95% CI 1.38–3.82), and 5-year mortality (HR 2.21 95% CI 1.64–2.98) (19). All models contained the following covariate set: age, sex, diabetes mellitus, insulin-treated diabetes mellitus, hypertension, hyperlipidemia, current smoker, previous smoker, prior myocardial infarction, prior percutaneous coronary intervention, concomitant coronary artery disease, and calendar year (19). Nonetheless, the main purpose of the study was a prognostic comparison among TTS, STEMI, and NSTEMI, and detailed information about other variables such as malignancies, pulmonary disease, TTS form, or triggers was not provided. Additionally, as recognized by the authors, their data did not allow them to assess the relative contribution of sex-related differences in the type of TTS stressor to the excess mortality observed in men (19). Our series, with a similar short-term mortality rate (3%) but a higher rate in the long term, included data from three countries, namely, Spain, Italy, and Germany. Thus, a country effect could not be discarded due to reporting differences, socioeconomic factors, rural–urban features, climate and other geographical factors, or regional healthcare policies.

Scarboro et al. performed a retrospective chart review of 210 TTS cases focusing on all-cause repeat hospital or emergency encounters. The authors remarked that standard medical therapy was not associated with protection and that tobacco smoking could lead to recurrent medical encounters with both cardiac and non-cardiac effects (20). In fact, in the same line, despite some prognostic TTS scores that have been previously published (GEIST, INTERTAK), a smoking habit is not included in any of them (2, 21).

However, while tobacco use increases oxidative stress, data in human cardiomyocytes modeling TTS also showed an increase in oxidative stress. It might be speculative that both TTS and concomitant smoking are relevant predictors of oxidative stress in cardiomyocytes and subsequently myocardial dysfunction (22).

Taken together, the data from the present study enforces the negative role of smoking in TTS, ruling out a “TTS smoking paradox” that suggests a positive influence on prognosis (23).

## Limitations

No cause-effect relationships can be established based on the present data due to the observational nature of the study, and further mechanistic investigations are needed. Smoking habit status during follow-up or the amount of tobacco consumed were not available and were not considered in the analysis. However, this study presents a large cohort with patient-level data on an infrequent disease managed in real-life settings, making it potentially the largest analysis on this topic to date.

## Conclusions

Our findings suggest that while smoking may influence the clinical presentation and course of TTS, it does not independently contribute to worse outcomes in terms of mortality. Further research is needed to better understand the complex relationship between smoking and TTS, including potential underlying mechanisms and long-term effects.

## Data Availability

The raw data supporting the conclusions of this article will be made available by the authors, without undue reservation.
